# “Swallowing the doctor”: an interview with João Conde about the future of nanomedicine

**DOI:** 10.1038/s42003-020-01293-6

**Published:** 2020-10-13

## Abstract

João Conde began his independent career at NOVA Medical School of Universidade Nova de Lisboa in the beginning of 2020. In this short Q&A he tells us about his experience as an early career researcher, challenges he faced with science under lockdown, the advice he has for his younger self and what is the most likely science fiction vision we can achieve with nanotechnology.

**Figure:**
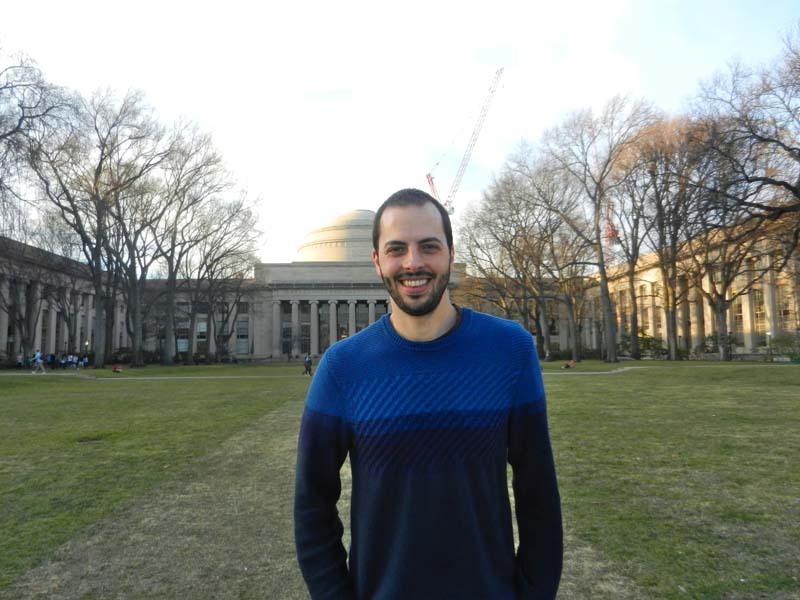
Catarina Conde

Can you tell us about your research interests?

The main focus of our research group is the use of cancer nanotechnology for precision medicine in order to tackle real and crucial medical problems involved in the development of novel and highly effective diagnostic and therapeutic platforms for cancer. Cancer has become the chief in providing groundbreaking platforms that can be used for precision medicine. Determining the response profile of a tumor, detecting key driver players in tumor progression, and trying to disable those drivers with targeted therapies and engineered materials so as to “smash” the brakes on malignant and metastatic cells to control proliferation is the *modus operandi* of our group.

Cancer nanotechnology is becoming a burgeoning field and our research brings up reality to the precision medicine initiative. Our research empowers the potential of nanomedicine to differentially combat cancer using smart and targeted platforms that mediate highly selective therapies within the tumor microenvironment. It is imperative to learn how advances in nanosystem capabilities are being used to identify new therapeutic tools driving the development of personalized medicine in different cancer types and disease states and recognize how to translate nanotechnology data and patients-derived Intel into an effective clinical strategy. The lack of standardized means to treat and profile the tumor microenvironment calls for a paradigm shift in the way we view and treat cancer. Is in this paradigm that our research focus: tackling real biomedical problems and develop smart materials to beat cancer.

Where did you find the initial spark for your interest in nanomedicine?

My initial spark for nanomedicine started when I read a transcript of the talk “Plenty of Room at the Bottom” by Richard P. Feynman in 1959 to the American Physical Society in Pasadena, which explores the immense possibilities afforded by miniaturization.

In this well-known talk, Feynman placed the theoretical foundations for the field now called nanotechnology when he conceived of a world in which things could be miniaturized, or huge volumes of information could be programmed onto smaller and smaller devices, and when machines could be made significantly reduced and compact. Despite the fact that 1959 was the time when computers were the size of entire rooms, Feynman asked his audience: “I don’t know how to do this on a small scale in a practical way, but I do know that computing machines are very large; they fill rooms. Why can’t we make them very small, make them of little wires, little elements, and by little, I mean little?”.

In fact, the concept of miniature medical minions isn’t new. Richard Feynman at that time already suggested the possibility of “swallowing the doctor”. Feynman told us “it would be interesting in surgery if you could swallow the surgeon (small machine). (…) It goes into the heart and looks around. It finds out which valve is the faulty one” and repairs it.

That truly inspired me to use nanomedicine as a powerful tool to diagnose and treat diseases like cancer.

What are your predictions for your field in the near future, given the underwhelming response of nanomedicines in clinical trials?

It is forecast that nanomedicine will definitely help move forward cancer treatments in patients, focusing on the combination of stratagems that are able to assemble converging therapy opportunities based on the “intel” from individual patients’ molecular signatures and innovative and efficient delivery vehicles.

Nevertheless, considering the extensive reports of anticancer nanomedicines in preclinical studies, we need to face the fact that there is a paucity of clinical trials using these therapies. We know that nanotechnology can certainly deliver, but we need to tackle the limitations that are holding back the translation of nanomedicines into the clinic and start benefiting from their full potential.

I believe that there is an earnest need to develop improved delivery vehicles and to establish guidelines regarding the performance metric by which we can evaluate a technology in a preclinical setting. Correlating preclinical and clinical outcomes would pave the way to generating a scoring system that would determine the probability of clinical success.

For successful translation into the clinic, an improvement in delivery efficiency must be realized through a mechanistic understanding of: (i) the characteristics of the tumor milieu; (i) the cancer response to a certain therapy; and (iii) the formation of criteria with which to assess therapeutic success based on a multifaceted metric that considers tumor size, survival rate, the nanomaterials’ pharmacokinetic/pharmacodynamic properties; and biodistribution, in order to enable correlations between preclinical and clinical settings.

From defeating the inconsistency in preclinical studies to enabling existing and broadly utilized systemic therapies with groundbreaking and proficient local platforms, one can influence existing and future studies to impart improved technologies with prognostic therapeutic performance. I believe the implementation of these guidelines can potentiate the development of a new generation of standardized drug delivery nanosystems.

Can you tell us about the challenges you had to face as a PI of a young lab due to the shutdown caused by the current pandemic? Has it affected the postdoctoral and graduate student funding and thus career development?

I remember perfectly well the day when the WHO Director-General declared the novel coronavirus outbreak a pandemic and a public health emergency of international concern. On this same day, the world changed unimaginably. I believe this is a historical moment as this new coronavirus changed life, every day, for all of us around the world.

And it was no different for our lab. Especially for a newly formed group, it was hard to stop all lab work and stay at home. Although this was necessary, it’s a fact that affected all of us and affected career development as we were unable to work in the lab for several months. However, this pandemic also brought us more time to think of future projects and new ideas to develop, and to write more opinion and review pieces.

Above all, this pandemic brought to all the understanding that science and medicine are more important than ever.

What advice would you give to your younger self?

The main advice I would give to my younger self is to be happy with what you do but at the same time to relativize things always. I believe this is the key to everything.

I always have this quote in mind from Marcel Pagnol, who was a French novelist, playwright, and filmmaker: “The reason people find it so hard to be happy is that they always see the past better than it was, the present worse than it is, and the future less resolved than it will be”.

Which is the most probable “science-fiction” scenario that you think could become reality, specifically with respect to cancer therapy?

I believe that idea from Richard Feynman represents that perfect “science-fiction” scenario that could become reality in cancer therapy: “swallowing the doctor” and having tiny machines that localize the error in cells and fix it. Achieving “the next big thing” starts with having these smart machines that could deliver drugs effectively to the most complex cell network – the tumor. In fact, the tumor microenvironment is a rough neighborhood for nanomaterials. Tumors create skewed neighborhoods, embedded with their leaky and chaotic blood vessels that are like broken roads or damaged sewers. All the different cells composing the tumor milieu actually shift to support tumor growth, these unoccupied lots densely overgrown with collagen fibers. And we are trying the get a huge truckload of medicines into all of this. Is not an easy task but I believe we are getting closer to building these tiny machines and to realizing Feynman’s “prophecy”.

*The editors at Communications Biology invite submissions in all areas of nanomedicine. For more details see* our recent editorial*on the subject.*

*This interview was conducted by Associate Editor Anam Akhtar*.

